# CRISPR-Cas12a (Cpf1): A Versatile Tool in the Plant Genome Editing Tool Box for Agricultural Advancement

**DOI:** 10.3389/fpls.2020.584151

**Published:** 2020-11-02

**Authors:** Anindya Bandyopadhyay, Nagesh Kancharla, Vivek S. Javalkote, Santanu Dasgupta, Thomas P. Brutnell

**Affiliations:** ^1^Reliance Industries Ltd., R&D-Synthetic Biology, Navi Mumbai, India; ^2^Chinese Academy of Agricultural Sciences, Biotechnology Research Institute, Beijing China; ^3^Gateway Biotechnology, Inc., St. Louis, MO, United States

**Keywords:** CRISPR, Cas9, Cas12a, NHEJ, base editing, PAM, temperature sensitivity, agriculture

## Abstract

Global population is predicted to approach 10 billion by 2050, an increase of over 2 billion from today. To meet the demands of growing, geographically and socio-economically diversified nations, we need to diversity and expand agricultural production. This expansion of agricultural productivity will need to occur under increasing biotic, and environmental constraints driven by climate change. Clustered regularly interspaced short palindromic repeats-site directed nucleases (CRISPR-SDN) and similar genome editing technologies will likely be key enablers to meet future agricultural needs. While the application of CRISPR-Cas9 mediated genome editing has led the way, the use of CRISPR-Cas12a is also increasing significantly for genome engineering of plants. The popularity of the CRISPR-Cas12a, the type V (class-II) system, is gaining momentum because of its versatility and simplified features. These include the use of a small guide RNA devoid of trans-activating crispr RNA, targeting of T-rich regions of the genome where Cas9 is not suitable for use, RNA processing capability facilitating simpler multiplexing, and its ability to generate double strand breaks (DSB) with staggered ends. Many monocot and dicot species have been successfully edited using this Cas12a system and further research is ongoing to improve its efficiency in plants, including improving the temperature stability of the Cas12a enzyme, identifying new variants of Cas12a or synthetically producing Cas12a with flexible PAM sequences. In this review we provide a comparative survey of CRISPR-Cas12a and Cas9, and provide a perspective on applications of CRISPR-Cas12 in agriculture.

## Introduction

Innovation has always been the driver of agricultural advancement from the earliest days of domestication to today’s machine learning-based genomic selection technologies. Although the green revolution provided the caloric increase to sustain the current global populations, this energy intensive form of agriculture is beginning to plateau (Food and Agriculture Organization of the United Nations, 2013). Future global agricultural production will depend increasingly on tools and technologies to improve sustainability and deliver more nutritious crops that will lessen our dependence of animal-based protein and deliver new fiber and plant medicinal products to market.

Unprecedented social and political resistance blocked the broad dissemination of genetically modified crops (GM crops), which has resulted in only a handful of traits being successfully introduced to the market. The precision, ease and low cost of engineered genomes using genome editing technologies promises to greatly reduce the technological and economic restrictions associated with Genetically Modified Organisms (GMOs), but public acceptance is by no means guaranteed (Smart et al., 2017; Callaway, 2018). Nevertheless, plant scientists from industry and academia around the globe have embraced the technology for numerous applications including gene knockouts, fine-tuning gene expression through transcriptional activation/repression, inducing epigenetic changes, multiplex gene editing, and base editing applications in crops. Importantly, the reagents for genome editing can be delivered into the cell without incorporating DNA into the genome (Svitashev et al., 2016; Zhang et al., 2016; Ma et al., 2020) and result in mutations that are identical to those occurring in nature, potentially simplifying the regulatory process associated with traditional GMO crops. Indeed, regulation itself is challenging with many genome editing events as it can be technologically challenging if not impossible to differentiate between a genome-edited change and one that occurs naturally. Consequently, editing by Clustered regularly interspaced short palindromic repeats (CRISPR) and CRISPR-associated proteins (Cas) is not only gaining popularity as a trait development tool, but also in achieving legal/regulatory approvals for product development in many countries (Schmidt et al., 2020).

Among clustered regularly interspaced short palindromic repeats (CRISPR) systems, Cas9 and Cas12a (originally identified as Cpf1) (Zetsche et al., 2015) have been most widely utilized and thus are most advanced in application. While the CRISPR-Cas9 system is still the most popular plant genome editing tool, the CRISPR-Cas12a nuclease is gaining broader adoption for multiple applications. One of the major limitations of CRISPR-Cas9 systems is the restriction of edits to regions of high GC content because of a “G” rich PAM sequence requirement (Bernabé-Orts et al., 2019). Although efforts are underway to engineer near “PAMless” Cas9 variants (Walton et al., 2020), only a few applications have been reported in plant systems (Zhong et al., 2019). Cas12a has the capacity to edit “T” rich PAM regions, and generates staggered ends that may promote site-directed integration events. Although the PAM sequence of Cas12a (e.g., TTTV) is longer than Cas9 (e.g., NGG), efforts have also been successful to engineer Cas12a variants with altered PAM specificities (Gao et al., 2017; Tóth et al., 2020). As recently shown in maize, Cas9 was used in combination with FLP recombinase to engineer gene stacks, greatly facilitating breeding efforts to stack traits of interest (Gao et al., 2020). This elegant use of genome editing technologies could be extended further by incorporating Cas12a, enabling a greater selection of target sites and potentially a higher efficiency of gene integration events. As promoters and introns are often AT-rich, the use of Cas12a also affords additional flexibility for engineering efforts (Wolter and Puchta, 2019). Although Cas12a displays a temperature sensitivity that has limited its utility in plant genome editing (Malzahn et al., 2019; Safari et al., 2019; Swarts, 2019), engineered variants have recently been generated with enhanced activities (Schindele and Puchta, 2020). In this review, we provide a comparison of CRISPR Cas9 and CRISPR Cas12a from the perspective of their applications in plant engineering and plant breeding and suggests ways to improve the utility of Cas12a in broadening its agricultural applications. Importantly, our intent is not to identify “the best” nuclease, but rather to highlight the advantages of broadening the molecular biology toolbox to incorporate both Cas9 and Cas12a technologies.

## The CRISPR-Cas System

The CRISPR-Cas system evolved as a bacterial immune system to combat the invasion of phages and other mobile genetic elements like plasmids and transposons (Hille et al., 2018). There are three major steps involved in the evolution of CRISPR-Cas systems in bacteria namely:

(i) CRISPR adaptation-integration of foreign invader genomic fragments into a CRISPR array as spacer sequences,
(ii) crRNA biogenesis – the CRISPR array is transcribed into pre-crRNA and processed to mature crRNA’s which in turn integrates with the Cas effector proteins to form crRNA effector complexes,
(iii) CRISPR interference – These programmed effector complexes identify and catalyze sequence-specific destruction of foreign invading genomic fragments (Jackson et al., 2017).

Based on the array of cas genes and the nature of the interference complex, the CRISPR-Cas system can be roughly divided into Class I and Class 2 systems which are further divided into six subtypes: Class I, type I, III, and IV are defined by multi-subunit complexes, and Class II, types II, V, and VI are postulated as single subunit effector endonucleases (Makarova et al., 2015; Shmakov et al., 2015). The Class 2 systems might have evolved from Class 1 systems with effector proteins originating from diverse mobile elements (Shmakov et al., 2015). Although Cas9 and Cas12-related proteins are similar in length (∼1100 to ∼1500 amino acids) it is likely that these families evolved independently from distinct transposable element families (Shmakov et al., 2015).

CRISPR-Cas systems have gained much popularity as a revolutionary genome-engineering tool because of their ease of use and multiple genome editing applications in the fields of medicine, agriculture, and animal husbandry. The most popular CRISPR-Cas9, originating from *Streptococcus pyogenes* (spCas9) belongs to the type II CRISPR system and has a protospacer adjacent motif (PAM) requirement of “NGG.” Here, we will focus on the development of the Cas12 system that offers distinct advantages for genome engineering.

### Cas12a, Class 2-Type V CRISPR System

The discovery and characterization of the Cas12a system was led by researchers at Broad Institute of MIT and Harvard University who identified a series of CRISPR nucleases in *Prevotella* and *Francisella 1* bacteria termed Cas12a (Cpf1) belonging to the Class 2, type V CRISPR system (Zetsche et al., 2015). Additional effector proteins of this family include Cas12b (C2c1) and Cas12c (C2c3), respectively. Cas12a is an endonuclease which varies in size between 1200 and 1500 amino acids (Shmakov et al., 2015). The PAM sequence requirement for Cas12a is “TTN/TTTN/TTTV”. (N = A/T/C/G; V = A/C/G). FnCas12a (from *Francisella novicida*), LbCas12a (from *Lachnospiraceae bacterium*), and AsCas12a (from *Acidaminococcus* sp.) are the three homologs of the Cas12a nucleases (Zetsche et al., 2015) (Table 1) which were widely utilized in plant genome editing technologies.

**TABLE 1:** List of various CRISPR-Cas12a nucleases and their various applications in crops.

Plant name	Cas12a	Gene codes	Gene targeted	Target Trait	Binary vector	Transformation method	PAM	References
Rice	FnCas12a	*OsDL, OsALS, OsNCED1, OsAO1*	*Drooping leaf; Acetolactate synthase; 9-cis-epoxycarotenoid dioxygenase1; Aldehyde oxidase*	Floral organ identity; Herbicide resistance; Abscisic acid regulation-stress tolerance; caroteniod catabolism and abscisic acid metabolism-stress tolerance	pPZP200	Agrobacterium	TTN	Endo A. et al., 2016
Rice	LbCas12a	*OsEPFL9*	Stomatal developmental gene	Abiotic stress tolerance	pCAMBIA	Agrobacterium	TTTG	Yin et al., 2017
Rice	LbCas12a	*OsPDS, OsBEL*	*Phytoene desaturase*; Bentazon-sensitive-lethal	Carotenoid biosynthetic pathway; Herbicide resistance	pHSN400	Agrobacterium	TTTA,	Tang et al., 2017
Rice	FnCas12a, LbCas12a	*OsBEL, OsRLK*	Bentazon-sensitive-lethal; Receptor like kinases	Herbicide resistance; Biotic stress stimulant response gene	pCambia	Agrobacterium	TTN, TTTG, TTTC	Wang et al., 2017b
Rice	FnCas12a, LbCas12a	*OsCAO1*	*Chlorophyllide-a oxygenase gene*	Photosynthetic efficiency	pUC19	Biolistic Mediated	TTTC	Begemann et al., 2017
Rice	AsCas12a, LbCas12a	*OsPDS, OsDEP1, and OsROC5*	*Phytoene desaturase*; Dense and erect panicle 1; Rice outermost cell-specific gene5	Carotenoid biosynthetic pathway; Regulating carbon-nitrogen metabolism-Yield; leaf rolling controlling; Negatively modulates bulliform cells	pYPQ203 or pYPQ202, pYPQ220, pYPQ230, pYPQ223	Agrobacterium	TTTG	Tang et al., 2017
Rice	FnCas12a	*OsDEP1, OsPDS, and OsEPFL9*	Dense and erect panicle 1; *Phytoene desaturase*; Stomatal developmental gene	Regulating carbon-nitrogen metabolism-Yield; Carotenoid biosynthetic pathway; Abiotic stress tolerance	pYPQ203	Protoplasts transformation	TTTC	Zhong et al., 2018
Rice	LbCas12a	*OsALS*	*Acetolactate synthase*	Herbicide resistance	pCXUN	Particle bombardment	TTTG	Li et al., 2018b
Rice	*FnCas12a, AsCas12a, and LbCas1*	*OsROC5 and OsDEP1*	Rice outermost cell-specific gene5; Dense and erect panicle 1	Leaf rolling controlling; Negatively modulates bulliform cells; Regulating carbon-nitrogen metabolism-Yield	pYPQ141, 210, 230	PEG-mediated protoplasts transfection	TTTC	Malzahn et al., 2019
Rice	LbCas12a	*OsDEP1, OsROC5*	Dense and erect panicle 1; Rice outermost cell-specific gene5	Regulating carbon-nitrogen metabolism-Yield; leaf rolling controlling; Negatively modulates bulliform cells;	STU-Cas12a system	Protoplasts transformation	TTTA	Tang et al., 2019
Rice	LbCas12a	*OsPDS, OsGS3, OsALS, OsNAL*	*Phytoene desaturase*; Grain size; *Acetolactate synthase*; Narrow leaf	Carotenoid biosynthetic pathway; Grain length-yield; Herbicide resistance; grain yield	STU-poly-A vector	Agrobacterium	TTTA, TTTG	Xu et al., 2019
Rice	AsCas12a, LbCas12a	*OsPDS*	*Phytoene desaturase;*	Carotenoid biosynthetic pathway;	pCAMBIA1301	Biolistic Mediated	TTTG	Banakar et al., 2020
Arabidopsis and rice	AsCas12a, LbCas12a	*OsPDS, OsDEP1, and OsROC5*	*Phytoene desaturase;* Dense and erect panicle 1; Rice outermost cell-specific gene5	Carotenoid biosynthetic pathway;	dAsCas12a–SRDX and dLbCas12a–SRDX carrying vector	Floral dip and protoplasts transformation	TTTG	Tang et al., 2017
Arabidopsis	LbCas12a, enLbCas12a, ttLbCas12a	five gene targets	–	–	enAsCas12a and ttLbCas12a carrying vector	Floral dip	TTTC, TTTA	Schindele et al., 2020
Soybean	LbCas12a/AsCas12a-RNP	*FAD2-1A, FAD2-1B*	*Fattyacid desaturase*	Increase oleicacid levels-Yield improvement	p2GW7	PEG-mediated protoplasts transformation	TTTTA	Kim et al., 2017
Tobacco	FnCas12a	*NtSTF1, NtPDS*	*Phytoene desaturase*; Stenofolia	NtPDS; NtSTF1	pRI201-AN	Agrobacterium	TTN	Endo A. et al., 2016
Tobacco	LbCas12a/AsCas12a-RNP	*AOC*	*Allen Oxidase Cyclase*	Jasmonic acid biosynthesis metabolism	p2GW7	PEG mediated RNP delivery	TTTN	Kim et al., 2017
Maize	LbCas12a	*Gl2*	Maize Glossy gene2	Epicuticular wax formation-Regulation of fattyacid elongase pathway	pYPQ141, 210, 230	Agrobacterium	TTTG, TTTC	Lee K. et al., 2019
Tomato	LbCas12a	*SlHKT1;2*	Salt tolerance gene 1;2	Salt tolerance-Abiotic stress tolerant	pHRHKT12.1	Agrobacterium	TTTG, TTTA	van Vu et al., 2020
Allotetraploid cotton	LbCas12a	*GhLCA1*	Cloroplastos alterados	Chloroplast developmental gene	pGhRBE3	Agrobacterium	TTTG	Li B. et al., 2019
Citrus	LbCas12a	*CsPDS*	*Phytoene desaturase;*	Carotenoid biosynthetic pathway;	p1380	Agroinfiltration	TTTC	Jia et al., 2019
Wheat	LbCas12a	*TaWaxy* and *TaMTL*	Waxy and Matrilineal	MTL-Haploid induction gene; Waxy- starch synthase gene involved in flour quality	pWMB110-LbCas12a	Agrobacterium	TTTG	Liu et al., 2020

### Structural Aspects of Cas12a

CRISPR-Cas12a is a two-component system, consisting of a protein/effector nuclease and a single crRNA. FnCas12a, LbCas12a, and AsCas12a proteins display similar domain organizations and range in size from ∼1300 to ∼1307 amino acids (aa). The crystal structure reveals a bi-lobed organization consisting of an α-helical recognition lobe (REC) and a nuclease lobe (NUC) (Dong et al., 2016; Yamano et al., 2016). The REC lobe consists of two domains Hel-1 and Hel-2, while the NUC lobe is comprised of the RuvC nuclease domain and three supplementary domains: PI, WED, and BH. The RuvC endonuclease domain of Cas12a is subdivided into three discontinuous segments (RuvC I–III), but it lacks the second HNH endonuclease domain and processes its mature crRNA without the utilization of trans-activating crispr RNA (tracrRNA) in comparison with Cas9 proteins (Safari et al., 2019; Wang J. et al., 2020).

**FIGURE 1:**
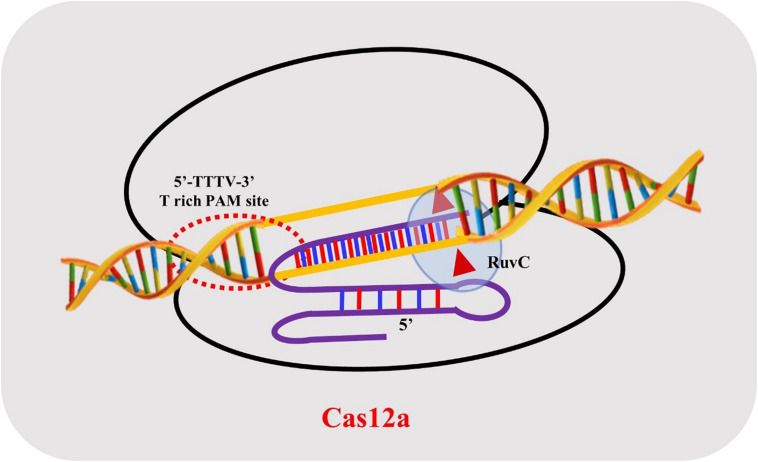
Schematic representation of Cas12a crRNA with the target strand DNA association.

**FIGURE 2:**
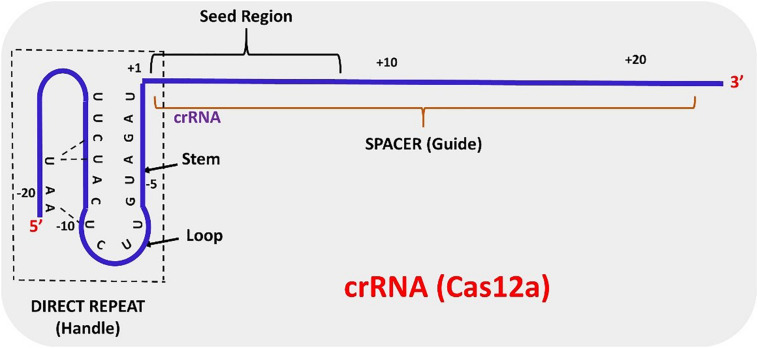
Schematic representation of mature crRNA derived from the maturation of pre-crRNA.

The structure of AsCas12a crRNA reveals 20 nt direct repeat (5’ handle) sequence and a spacer (guide segment) sequence of 23 nt in length (Figure 1). The crucial pseudoknot structure adopted by the direct repeat sequence is essential for the recognition by Cas12a. The pseudoknot structure can be broadly divided into a stem and a loop region. The pseudoknot, starting from -1 to -20 bases, consists of five Watson-Crick base pairs, one noncanonical U–U base pair, one UCUU tetraloop, one reverse Hoogsteen A–U base pair and three 5′-end bases (Figure 2). The hydrogen bonds formed within stem and loop regions stabilizes the pseudoknot structure. The bases U (-1), U (-10), U (-16), and A (-18) are conserved across Cas12a homologs indicating formation of similar tetraloop pseudoknot is crucial for the efficiency of endonuclease activity of Cas12a. The guide segment (spacer) sequence is complementary to the target DNA sequence and seed sequences (1–8 bases) are crucial for target specificity of CRISPR-Cas12a system (Dong et al., 2016; Yamano et al., 2016; Li L. et al., 2018; Swarts and Jinek, 2018; Safari et al., 2019). A survey of 16 uncharacterized Cas12a enzymes revealed differences in both PAM recognition and cut site repair mechanism (Zetsche et al., 2019), suggesting that it should be possible to engineer a range of activities into members of the Cas12a family.

### crRNA Biogenesis

In contrast to CRISPR-Cas9 systems, type V systems do not require tracrRNA and RNase III for processing of mature crRNA. The transformation of pre-crRNA to mature crRNA (42–44 nt length) is mediated by intrinsic ribonuclease activities of Cas12a domains. The biogenesis of mature crRNA in *F. novicida* starts with the recognition of 27–32 base pair (bp) long spacers located adjacent to 36 bp long repeats by FnCas12a which are expressed as a single transcript (Zetsche et al., 2015). The repeat sequences in the pre-crRNA transcript forms a pseudoknot structure which is readily recognized by Cas12a (Dong et al., 2016; Yamano et al., 2016; Swarts and Jinek, 2018). Pseudoknot binding to divalent cations like Mg^2+^ or Ca^2+^ augments binding of the crRNA to Cas12a. The WED domain of Cas12a catalyzes the processing of the 5′ end of the crRNA but the 3′ end processing mechanism of crRNA is still obscure. A mature crRNA consists of 19–20 nt direct repeat sequence (5′ pseudoknot structure) and 20–24 nt guide or spacer sequence (Swarts and Jinek, 2018; Safari et al., 2019) (Figure 2).

### Salient Difference Between Cas12a and Cas9

**FIGURE 3:**
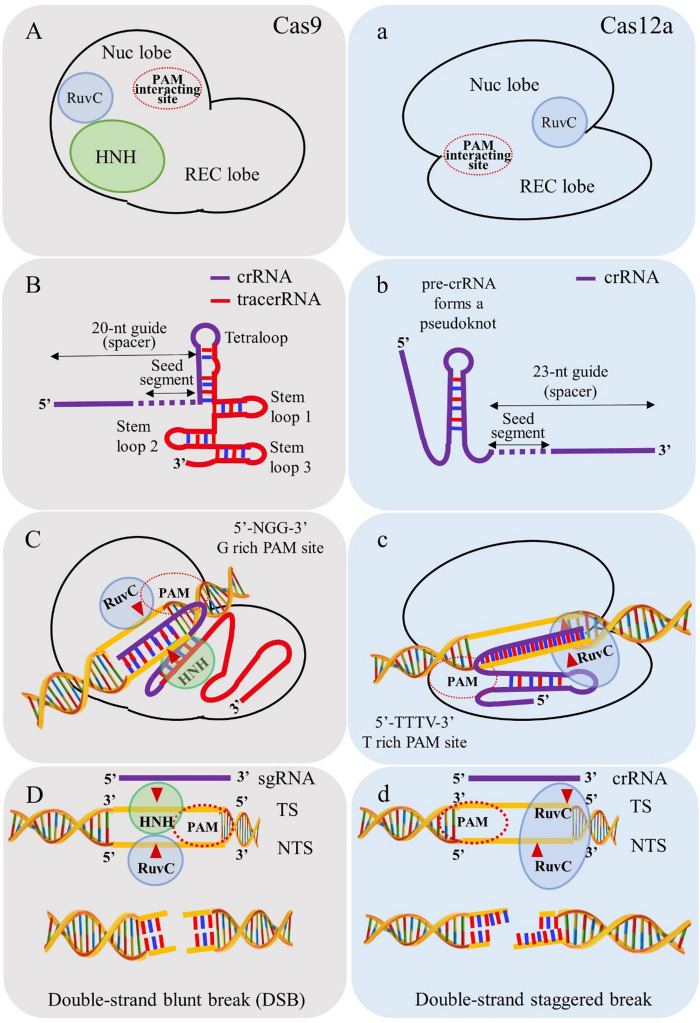
Depiction of salient differences between Cas9 and Cas12a. **(A)** Cas9 contains two endonuclease domains to cleave target strand (TS) and non-target DNA strands (NTS) by HNH and RuvC domains, respectively. **(B)** Cas9 requires tracrRNA for biogenesis of mature crRNA. **(C)** PAM requirement of Cas9 is “NGG” rich regions for cleaving target site. **(D)** Cas9 simultaneously breaks TS and NTS and generates blunt ends. **(a)** Cas12a utilizes single endonuclease domain RuvC for cleaving TS and NTS. **(b)** Cas12a processes its own mature crRNA without intervention of tracrRNA. **(c)** PAM requirements of Cas12a is “TTN/TTTN” favoring “AT” rich regions. **(d)** Cas12a cleaves in a sequential manner in which NTS is cleaved first and followed by TS and generates double strand staggered break (sticky ends).

The major differences between Cas9 and Cas12a proteins (see Figure 3) include the following:

(i) The PAM requirement for Cas12a is “TTTN” which favors its use in targeting “AT” rich regions in the genome in contrast to the spCas9 system (PAM “NGG”) (Zetsche et al., 2015);
(ii) Cas12a cleaves the target DNA strand 18-23 nucleotide (nt) distal of the PAM, leaving staggering ends (5 to 8 nt 5’ overhangs) in contrast to blunt ends generated by Cas9 (Zetsche et al., 2015);
(iii) Cas12a processes the mature crRNA into 42 to 44 nt segments in contrast to Cas9 requiring tracrRNA for biogenesis of mature crRNA. This distinct feature of Cas12a makes it advantageous for multiplex gene editing, transcription, epigenetic modulations and base editing (Safari et al., 2019);
(iv) Unlike Cas9, Cas12a contains only one endonuclease domain, RuvC (NUC lobe) for cleavage of target and non-target DNA strands. The cleavage occurs in a sequential manner in which the non-target DNA strand is cleaved first and later the target DNA strand by the RuvC domain (Yamano et al., 2016);
(v) Lower off-target effects have been reported for Cas12a relative to Cas9, that are indistinguishable from spontaneous mutations caused during plant development (Bernabé-Orts et al., 2019). However, it has also been reported that Cas12a and several orthologs are capable cleaving randomized targets *in vitro* that contain up to four mismatches (Murugan et al., 2020). It remains to be seen, however, if this reduced specificity is evidenced in planta;
(vi) One of the major constraints of Cas12a broader adoption in plants is its lower efficiency at low temperatures (Malzahn et al., 2019);
(vii) A modified dCas12a fused to a human apolipoprotein B mRNA editing enzyme did not activate the DNA damage response and increased deamination efficiency and editing specificity relative to a similar dCas9 base editor nickase (Wang X. et al., 2020).
(viii) Intellectual property issues of Cas9 invention rights between the University of California vs Broad Institute of MIT and Harvard are still obscure and disorganized whereas Cas12a patent rights are a single point grant to Broad institute, MIT, and Harvard.

## Application of CRISPR-Cas12a in Agriculture

Cas12a editing has been widely utilized in many crops (see Table 1) including rice (Endo A. et al., 2016; Begemann et al., 2017; Hu et al., 2017; Tang et al., 2017, 2018; Wang et al., 2017a, b; Yin et al., 2017; Li L. et al., 2018; Li et al., 2019a; Jun et al., 2019; Malzahn et al., 2019; Banakar et al., 2020; Chen et al., 2020; Schindele and Puchta, 2020), wheat (Liu et al., 2020), maize (Lee K. et al., 2019), soybean (Kim et al., 2017), cotton (Li B. et al., 2019), tomato (van Vu et al., 2020), citrus (Jia et al., 2019), tobacco (Endo A. et al., 2016; Endo and Toki, 2019), and the model plant Arabidopsis (Wolter and Puchta, 2019; Schindele and Puchta, 2020). At present, three Cas12a genome editing systems AsCas12a, FnCas12a, and LbCas12a have been demonstrated in plants (Zhong et al., 2018) with varied efficiency.

Rice is one of the most well-studied crops due to its agricultural importance, small genome size, ease of transformation and available genetic resources making it an ideal flagship genome for the grasses (Mishra et al., 2018). These factors have also made it an ideal testing ground for developing genome editing technologies. Codon optimized FnCas12a binary vectors were utilized for targeted mutagenesis in rice (*OsDL, OsALS, OsNCED1, OsAO1*) and tobacco (*NtPDS and NtSTF1*) with average targeted mutation frequencies of 47.2% and 28.2%, respectively (Endo A. et al., 2016). Utilizing the LbCas12a nuclease two endogenous rice genes *OsPDS* and *OsBEL* were targeted with mutation frequencies of 21.4 and 41.2%, respectively (Xu et al., 2017). An independent study that targeted the disruption of *OsPDS* by LbCas12a resulted in a similarly high editing frequency of 32.3% (Banakar et al., 2020). Xu et al., 2017 also demonstrated that pre-crRNAs were more efficient in generating mutants than mature crRNAs in rice. However, the opposite was observed in HEK293T cells (Zetsche et al., 2017). In addition to these proof-of-concept experiments, LbCas12a was also used to create loss-of-function alleles of *OsEPFL9* which regulates stomatal density. These lines increased water use efficiency eight fold in T2 generation plants (Yin et al., 2017).

*In vitro* experiments conducted with FnCas12a and LbCas12a suggests that the efficiency of Cas12a depends upon the base content of the gene sequence targeted (Wang et al., 2017b). To compare the activity of two nucleases, *Acidaminococcus* sp. BV3L6 (As) and *Lachnospiraceae bacterium* ND2006 (Lb) were used to target six sites in three genes (*OsPDS, OsDEP1*, and *OsROC5*). Mutation frequencies observed ranged from 0.6 to 10% for AsCas12a and 15–25% with LbCas12a across the six targets (Tang et al., 2017). Importantly, whole-genome sequencing analysis of LbCas12a-edited plants revealed zero off-target mutations in the rice genome (Tang et al., 2018).

A potential advantage in using Cas12a in genome editing, is its ability to facilitate site-directed integration due to staggered overhangs. The expression of *F. novicida* and *L. bacterium ND2006* nucleases resulted in a high frequency of homology-directed repair (HDR) in rice suggesting a primary advantage of the Cas12a system over Cas9 for targeted gene integration (Begemann et al., 2017). However, the FnCas12a mutation frequency varies with the selection of PAM sequence (e.g. 10% to 35% efficiency with “TTTV” and 5–10% with “TTV”) in rice (Zhong et al., 2018), suggesting that site directed targeting of sequences may be highly variable across the genome.

Given the inherent variations in site-specific editing efficiencies, it is challenging to directly compare the mutation efficiencies of Cas9 and Cas12a. Although studies in several plant species have suggested lower editing efficiencies associated with Cas12a relative to Cas9 (Lee K. et al., 2019; Malzahn et al., 2019; Liu et al., 2020), Wang and colleagues used Cas9 and Cas12a to target the same loci and in one instance observed a higher efficiency of mutation with Cas9 (Lee K. et al., 2019) and with another target Cas12a was more efficient (Banakar et al., 2020). Various factors which might have attributed to the relative efficiency could be related to the gRNA sequences, epigenetic modifications of the target site or expression of the endonuclease itself. Despite the variation in editing efficiencies, several groups have utilized Cas12a to edit more recalcitrant genomes including the allotetraploid cotton (Li B. et al., 2019), citrus (Jia et al., 2019), soybean (Kim et al., 2017) and wheat (Liu et al., 2020). Thus, although it appears that Cas12a is generally less efficient, as discussed below, newly developed versions of Cas12a promise to enhance its performance *in planta*.

## Future Perspective: Improving Cas12a for Greater and Broader Applications in Agriculture

Genome editing has opened up tremendous opportunities to improve the pace of agricultural advancement. The EU was one of the first organizations to develop a regulatory framework for genome editing (Friedrichs et al., 2019). They defined three tiers of genome editing. Site-directed nuclease (SDN) 1 events are the result of non-homologous end-joining and result in single nucleotide changes or small indels. SDN2 events result in template-mediated changes of a few nucleotides. In rice, for example, herbicide-resistant mutant lines were developed using template-mediated repair. LbCas12a was used create staggered breaks in the *Acetolactate synthase* (*ALS*) gene in the presence of a template molecule containing the point mutations of interest. Repair through a HDR mechanism (Li et al., 2018a) resulted in the precise introduction of small nucleotide changes. Although herbicide tolerance has been achieved effectively in major crop plants through transgenic approaches, the reduced regulatory barriers associated with CRISPR-mediated edits (especially outside the European Union, e.g., United States and Australia) makes this trait an attractive target for species that have had limited success overcoming regulatory hurdles with transgenic technologies such as rice. CRISPR genome edited crops may be exempted from GMO regulation in several countries on a case-by-case review including the United States, Canada, Australia, Japan, Colombia, Brazil, Argentina, and Chile. Other countries including the United Kingdom, India, Bangladesh, Philippines, and Indonesia are still in the process of developing regulations while the European Union and New Zealand have classified genome-edited crops as GMO’s as they consider the process rather than the outcome. Events that insert foreign DNA from another species are likely to trigger the most rigorous regulatory reviews (SDN3) and will likely be considered transgenic in most countries. In the United States, the Environmental Protection Agency (EPA), the United States Department of Agriculture (USDA) and the Food and Drug Administration (FDA) all influence the regulatory path of an engineered plant product. Thus, it is still challenging to predict the time and costs of bringing genome-edited products to market (Schmidt et al., 2020).

Despite the challenges of the current regulatory environment, the scope and scale of genome editing opportunities will likely drive the entire agricultural industry. A few examples that incorporate genome editing technologies include accelerated breeding strategies (Li et al., 2016; Chen et al., 2019), allelic replacements (Ahmar et al., 2020), simultaneous double haploid production and editing (HI-EDIT) (Kelliher et al., 2019; Wang et al., 2019), crop domestication (Van Tassel et al., 2020; Zhang et al., 2020), and gene stacking (Razzaq et al., 2019). Importantly, these technologies are enabling a step change in the pace of crop improvement over conventional breeding and transformation technologies, especially when combined with emerging transformation technologies (e.g., co-expression of maize transcriptional factors, *BABY BOOM* and *WUSCHEL2*) (Lowe et al., 2016), machine learning (e.g., breeding), and imaging technologies. With so many potential opportunities, it is clear that both Cas9 and Cas12a will be utilized extensively in plant breeding in the years ahead. Nevertheless, several technological improvements in Cas12a will help to accelerate its broader adoption and utility.

**FIGURE 4:**
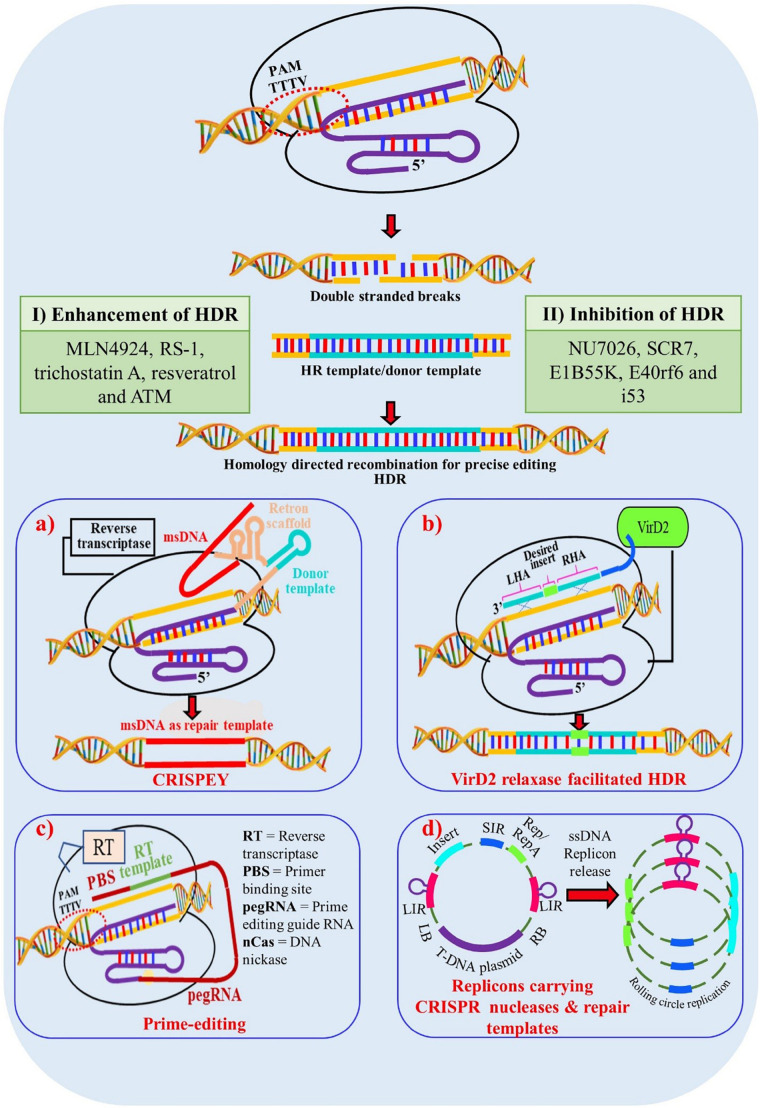
Enhancement of Homology Directed Recombination (HDR) through multiple approaches. **(I)** Addition of chemical components which enhances HDR mechanisms in cells; **(II)** Chemical components which inhibit non homologous end joining (NHEJ) and thus indirectly promote HDR mechanism in cells; **(a)** Enhancement of HDR through CRISPEY (Cas9 Retron precISe Parallel Editing via homologY) method. Utilization of bacterial retron system to generate desired single stranded donor DNAs via multi-copy single-stranded DNA (msDNA); **(b)** Enhancement through VirD2 relaxase gene. A chimeric protein is synthesized with Cas9 protein tethered to the Agrobacterium VirD2 relaxase protein. Cas9 generates a precise DSB and VirD2 relaxse brings the donor template into close proximity to the DSB; **(c)** HDR enhancement through prime-editing for precise genome editing for crop improvement. **(d)** Enhancement through geminiviral replicon system. Utilization of rolling circle mechanism of geminivirus replicon system to generate multiple donor templates in vivo to enhance the success of HDR; msDNA – multi-copy single-stranded DNA; LHA – Left Homologous Arm; RHA – Right Homologous Arm; LB – Left Border; RB – Right Border; LIR – Long Intergenic Regions; SIR – Short Intergenic Regions.

Although, Cas12a has many advantages there are certain limitations as well which need to be addressed, such as PAM flexibility, to enable its broader application across the genome. Greater extent of modified or engineered versions of Cas12a are needed for single gene or multigene activation or repression. The relatively high temperature dependence of Cas12a is problematic in plant transformation as many crop protocols require low temperature. A Cas12a nickase has not been engineered to date which could facilitate gene integration without DSB, improving the possibility of HDR for allele replacement (Figure 4).

### Development of Cas12a With Relaxed or “PAM-Less” Requirements

One of the significant limitations of Cas12a in plant genome editing is the relatively long TTTV PAM sequence requirements in eukaryotic genomes (Tóth et al., 2020). The theoretically estimated frequency of the TTTV PAM motif in DNA sequences is 3/256, a considerably more restrictive target space than that of the canonical NGG motif of SpCas9 (16/256). Several groups have recently attempted to engineer alternative PAM site requirements into Cas12a (Table 2). Perhaps the most promising is the improved Cas12a variant (*impLbCas12a*) generated by Toth and colleagues (Tóth et al., 2020). After engineering five independent nucleotide changes into Cas12a that had been shown in previous studies to modulate PAM-site selectivity and enzyme cutting efficiency, the *impLbCas12a* enzyme was able to cut at a TNTN consensus sequence with increased activity (Tóth et al., 2020). In addition to engineering relaxed specificities, Chen and colleagues identified two Cas12a variants (*CeCas12a* and *BeCas12a*) with a more stringent PAM site requirement in order to minimize off target events (Chen et al., 2020). This may have applications in engineering synthetic circuits when tight control of target sites is necessary. To continue expanding the repertoire of PAM sites or enhance enzyme activity, technologies such as phage-assisted continuous evolution (PACE) have shown much promise (Esvelt et al., 2011; Komor et al., 2016). In addition, the exploration of Cas12a homologs in diverse bacterial species, such as Lb5Cas12a, BoCas12a, BsCas12a (e.g., Zetsche et al., 2015; Gao et al., 2017) will likely provide both novel insights into Cas12a function and potentially new activities. However, it is important to note that all of these advances have come from studies in non-plant systems, thus considerable work remains to test these functionalities and develop new variants specifically for plant systems.

**TABLE 2:** List of modified and native Cas12a nucleases with their flexible “PAM” specificities.

CRISPR/Cas12a system	Modification in native CRISPR/Cas12a	PAM specificity	Amino acids Size	References
FnCas12a	Native	TTV,TTTV,KYTV	1300	Zetsche et al., 2015
FnCas12a-RR	N607R/K671R	TYCV, TCTV	1300	Toth et al., 2018; Zhong et al., 2018; Tóth et al., 2020
FnCas12a-RVR	N607R/K613V/N617R	TWTV	1300	Toth et al., 2018; Zhong et al., 2018; Tóth et al., 2020
FnCas12a-RVRR	N607R/K613V/N617R/K671R	TYCV, TCTV, TWTV	1300	Tóth et al., 2020
AsCas12a	Native	TTTV	1307	Zetsche et al., 2015; Kim et al., 2017; Tang et al., 2017; Bernabé-Orts et al., 2019
AsCas12a-RR	S542R/K607R	TYCV,CCCC	1307	Gao et al., 2017
AsCas12a-RVR	S542R/K548V/N552R	TATV	1307	Gao et al., 2017
AsCas12a-RVRR	S542R/K548V/N552R/K607R	TYCV,CCCC,TATV	1307	Tóth et al., 2020
LbCas12a	Native	TTTV	1228	Zetsche et al., 2015; Kim et al., 2017; Tang et al., 2017; Bernabé-Orts et al., 2019; Li et al., 2019b
LbCas12a-RR	G532R/K595R	TYCV,CCCC	1228	Gao et al., 2017; Li et al., 2018a; Zhong et al., 2018; Tóth et al., 2020
LbCas12a-RVR	G532R/K538V/Y542R	TATV	1228	Gao et al., 2017; Li et al., 2018a; Zhong et al., 2018; Tóth et al., 2020
LbCas12a-RVRR	G532R/K538V/Y542R/K595R	TYCV,CCCC,TATV	1228	Tóth et al., 2020
MbCas12a	Native	TTV, TTTV	1373	Zetsche et al., 2015
MbCas12a-RR	N576R/K637R	TYCV, TCTV	1373	Toth et al., 2018
MbCas12a-RVR	N576R/K582V/N586R	TWTV	1373	Toth et al., 2018
MbCas12a-RVRR	N576R/K582V/N586R/K637R	TYCV, TCTV, TWTV	1373	Tóth et al., 2020
enAsCas12a	E174R/S542R/K548R	VTTV,TTTT,TTCN,TATV	1307	Kleinstiver et al., 2019
impLbCas12a	D156R, G532R, K538V, Y542R, k595R	TTTV,TCCV, CCCV, TATC, TACV	1228	Tóth et al., 2020

### Cas12a for Gene Expression Modulation

As mentioned above, Cas9 has been used to manipulate gene expression through the engineering of DNAse dead (dd) enzymes that are directed to specific sites in the genome and similar strategies are now ongoing to engineer Cas12a (Table 3). The dual nuclease activity of Cas12a is essential for its ability to create double strand breaks (DSB) in the DNA and is dependent on the RuvC domain (Zhang et al., 2017; Safari et al., 2019). Alteration or mutation of the RuvC domain leads to the formation of a DNAse dead Cas12a (dCas12a) which retains the crRNA processing activity of Cas12a but fails to cleave the DNA (Zetsche et al., 2015). To exploit this feature of Cas12a, Leenay and colleagues created catalytically inactive ddCas12a enzymes to identify the repertoire of PAM sites in an *in-vivo* screen and infer binding specificity based on green fluorescent protein (GFP) readout (Leenay et al., 2017). Transcriptional repression mediated through both the inhibition of elongation and initiation in *E.coli* was achieved when a ddCas12a was targeted to multiple promoter or coding sequences (Zhang et al., 2017). A similar strategy was used to create a ddCas12a variant for *Streptomyces*, an important bacterial species for natural product discovery (Li L. et al., 2018). Additional modifications of Cas12a applied to human (Gao et al., 2018) and *Escherichia coli* (Miao et al., 2019) systems, have increased the activity of DNAse and activation/repression modalities of Cas12a. Although it is possible that similar mutations would also be effective in plant systems, this has yet to be demonstrated. In Arabidopsis, Tang et al. (2017) demonstrated transcriptional repression of *miR159b* utilizing deactivated nuclease domains of dAsCas12a (D908A) and dLbCas12a (D832A). Although, AsCas12a performed better than LbCas12a as a transcriptional repressor in *Arabidopsis thaliana*, species-specific differences are likely to influence binding efficiencies. Thus, further improvement in this area is greatly needed in plants, as LbCas12a is still the most widely utilized editing tool among all of the Cas12a variants, but reports in crop plants are relatively limited.

**TABLE 3:** List of Cas protein activators and repressors and their applications in inducing gene expression in plants and human embryonic cells.

Activator/Repressor	Target Gene/Plant species	References
denAsCas12a–VPR	human cells:S170R, E174R, S542R or K548R	Kleinstiver et al., 2019
enAsBE1.1–1.4	human cells:S170R, E174R, S542R or K548R	Kleinstiver et al., 2019
dAs/LbCas12a-VP64-3xHA-crHDV	Luciferase (luc) gene in HEK293T cells	Gao et al., 2018
dCas9-H3K27 acetyltransferase p300	Flowering locus in Arabidopsis	Lee J. E. et al., 2019
dCas9-H3K9 methyltransferase KRYPTONITE	Flowering locus in Arabidopsis	Lee J. E. et al., 2019
dCas9-VP64	Flowering locus in Arabidopsis	Lee J. E. et al., 2019
dCas9-SRDX (Transcriptional repressor)	Flowering locus in Arabidopsis	Lee J. E. et al., 2019
dCas9-H3K9 methyltransferase G9a	Flowering locus in Arabidopsis	Lee J. E. et al., 2019
dCas9-MS2-VP64	Rice	Lowder et al., 2018
dCas9-mTALE-VP64	Rice	Lowder et al., 2018
dCas9–6TAL–VP128 (dCas9-TV)	Protoplasts of Arabidopsis	Li Z. et al., 2017
dCas9-VP128	Protoplasts of Arabidopsis	Li Z. et al., 2017
dCas9–VP256	Protoplasts of Arabidopsis	Li Z. et al., 2017
dCas9-VPR	Human cells- HEK293T	Chavez et al., 2016
dCas9-SAM	Human cells- HEK293T	Chavez et al., 2016
dCas9-SunTag	Human cells- HEK293T	Chavez et al., 2016

### Cas12a Efficiency Improvement Through Chemical and Engineering Modifications

In addition to engineering the Cas12a protein, several groups have tried to optimize Cas12a cutting efficiencies and reduce off target modifications by modifying the crRNA molecule and Cas12a transcript. Li and colleagues showed in human cell lines by engineering a crRNA molecule containing five 2’-fluoro ribose at the 3’ terminus together with an engineered Cas12a mRNA template in which uridine residues were replaced with pseudouridine throughout the entire transcript, cutting efficiencies could be improved. Together, these modifications led to an enhanced cutting efficiency of 300% above the wild-type plasmid template and crRNA controls (Li B. et al., 2017). Extensions of the crRNA at the 5’end also improved the efficiency of Cas12a NHEJ and HDR activities (Park et al., 2018). Bin Moon et al., 2018 also demonstrated precise and enhanced indel-generating efficiency of Cas12a, up to 13-fold, with an engineered 3’-uridinylate rich crRNA in human HEK-293T (Human Embryonic Kidney) cells. This may be due in part to the enhanced stability of the molecule especially when Cas12a and crRNA are delivered to the cell as an ribo nucleo protein (RNP) in primary mouse myoblasts (Park et al., 2018).

McMahon et al., 2018 also demonstrated in HEK-293T cells that truncated synthetic RNA’s (scrRNA) with chemical modification of nucleotides at 5’ and 3’ end with PS, 2’-F’5’-O-Me, and substitution with DNA nucleotides were more readily taken up by cells and enhanced its genome editing efficiency of AsCas12a relative to wild-type crRNAs. To identify additional components that may aid editing efficiencies, Ma and colleagues conducted a small molecule library screen and identified VE-822 and AZD-7762 for their ability to enhancing the genome editing efficiency of Cas12a in human pluripotent stem cells (Ma et al., 2018). In summary, chemical modification to the crRNA, Cas12a transcript and the addition of small molecules all were able to improve Cas12a efficiencies in mammalian systems. It remains to be seen, however, if any of these modifications result in similar efficiencies in plant systems. Some of the challenge in introducing chemical modifications could be overcome in plants if they are transformed using biolistics as it is easier to envision an RNP cocktail with small molecules rather than utilizing Agrobacterium transformation.

### Improving HDR Efficiency

The DSBs generated by site-specific nucleases (SSNs) are repaired broadly through two repair pathways; non-homologous end joining (NHEJ) or homology directed repair (HDR) methods, generating either random or directed outcomes. In higher organisms and especially in plants the preferred DSB repair mechanism is achieved through NHEJ, where most often small indels are created causing frameshift mutations ultimately creating loss-of-function or “knock-out” alleles. If a DNA template (either single or double stranded) is present when the DNA is cleaved, then DNA repair can be achieved through HDR. This mechanism results in precise gene editing or modifications which is the most preferred way of bringing changes in the plant genome to produce high yielding and disease-resistant varieties in the crops. Unfortunately, the efficiency/frequency of HDR in plants is very low due to numerous factors including the low copy number of donor templates and length of donor templates (Puchta, 2005). Several groups have targeted the DNA repair pathway to engineer higher efficiencies of HDR including the targeted suppression of KU70 and KU80 and the overexpression of RAD54, RAD51, CtIP, CDK1, and Scr7 inhibitor to bypass NHEJ and promote HDR pathways (Shaked et al., 2005; Bozas et al., 2009; Qi et al., 2013; Maruyama et al., 2015; Ye et al., 2018; Table 4). These studies reveal that overexpression of HDR-related factors and suppression of NHEJ related factors are promising approaches to homology-directed gene targeting-HGT (Pinder et al., 2015; Robert et al., 2015; Endo M. et al., 2016; Rozov et al., 2019). The utilization of a geminivirus replicon system also enhances donor template delivery and available donor templates enhance homologous recombination (Baltes et al., 2014). However, this system failed to generate a high HDR repair frequency in Arabidopsis, suggesting species specific variation (De Pater et al., 2018; Hahn et al., 2018). A combination of an Agrobacterium VirD2 relaxase gene with Cas9 improved HDR in rice presumably by enhancing the proximity of repair template to the DSBs in rice (Ali et al., 2020). *In planta* gene targeting is another efficient method of HDR which was successfully demonstrated in rice (Sun et al., 2016), maize (Kumar et al., 2016) and Arabidopsis (Fauser et al., 2012; Schiml et al., 2014; Hahn et al., 2018; Wolter and Puchta, 2019). Here, sequence specific nucleases not only generate targeted DSB but also release a homology template from the T-DNA backbone. Another innovative method to increase HDR that has been applied in yeast systems is CRISPEY (Sharon et al., 2018). In this system, a DNA retron is used to tether a template sequence to a gRNA that is then delivered to the genomic target. Although the efficiency of HDR is high in yeast, it remains to be seen if a similar approach will work in plants. Prime editing can be used to introduce precise point mutations and insertions in the plant genome without separate repair templates and with reduced off-targets compared to other genome editing technologies (Anzalone et al., 2019). However, inherent limitations associated with prime editing must still be overcome if it is to be broadly adopted as an efficient, precise and flexible plant genome editing tool for crop improvement (Lin et al., 2020; Marzec and Hensel, 2020; Xu et al., 2020).

**TABLE 4:** List of selected Cas9 and Cas12a homology-directed gene targeting mechanisms and their efficiencies in crops and animals.

Nuclease	HDR Enhancing System	Target Gene	Target Organism/plant	HDR efficiency percentage	References
FnCas12a and LbCas12a	Repairing DNA template flanked by 1000 bp homology DNA fragments/arms	Chlorophyllide-a oxygenase gene (CAO1)	Rice	3-8	Begemann et al., 2017
LbCas12a	Ribonucleo Protein (RNP) Complex	*slc45a2(albino) and tyr (tyrosinase)*	Zebrafish and Xenopus	5-7	Moreno-Mateos et al., 2017
	Synthesis dependent repair with donor repair template coupled with left homologous arm is sufficient for HDR mechanism	Acetolactate synthase gene (ALS)	Rice	0.014	Li et al., 2018a
				0.021	
	Ribozyme based strategy to synthesize crRNA’s and DNA repair template	Acetolactate synthase gene (ALS)	Rice	4.6	Li et al., 2019a
				1.7	
	Utilization of homologous recombination enhancers	Acetolactate synthase gene (ALS)	Arabidopsis	1.47	Wolter and Puchta, 2019
	Utilization of viral multi replicon system (de novo engineered geminiviral replicon system) to increase the availability of donor template	Salt-tolerant (SlHKT1;2)	Tomato	4.5 - 9.8	van Vu et al., 2020
CRISPR-SpCas9	SpCas9, sgRNA and single-stranded DNA oligo’s (72 base pair) into plant cells	Phytoene desaturase-OsPDS	Rice	6.8	Shan et al., 2013
CRISPR-Cas9	gRNAs targeting Lig4 gene were transformed with Cas9- Lig4 knockout resulted in enhancement of HDR	Acetolactate synthase gene (ALS)	Rice	0.15-1.0	Endo M. et al., 2016
	Geminivirus based vectors to release abundant HDR template	Actin-1 (ACT1)	Rice	6.8 - 19.4	Wang et al., 2017a
		Glutathione S-transferase (GST)		7.7	
CRISPR-SpCas9	DNA donor template contains constitutively expressing PAT gene with 1 kb homologous arm surrounding the target gene	Liguleless1-LIG1	Maize	0.2 - 4.61	Svitashev et al., 2015
		Male fertility genes - Ms26		0.13 - 3.11	
		Male fertility genes - Ms45		0.47 - 1.87	
		Acetolactate synthase gene (ALS1 and ALS2)		1.35 - 2.23	
CRISPR-Cas9-VirD2	Cas9-VirD2 chimeric protein helps in DSB and bringing close proximity of phosphorothioate mediated template DNA through VirD2 relaxase	Acetolactate synthase gene (ALS)	Rice	4.1 - 20.8	Ali et al., 2020
		Histone Deactylase (HDT)		0.2 - 8.7	

**FIGURE 5:**
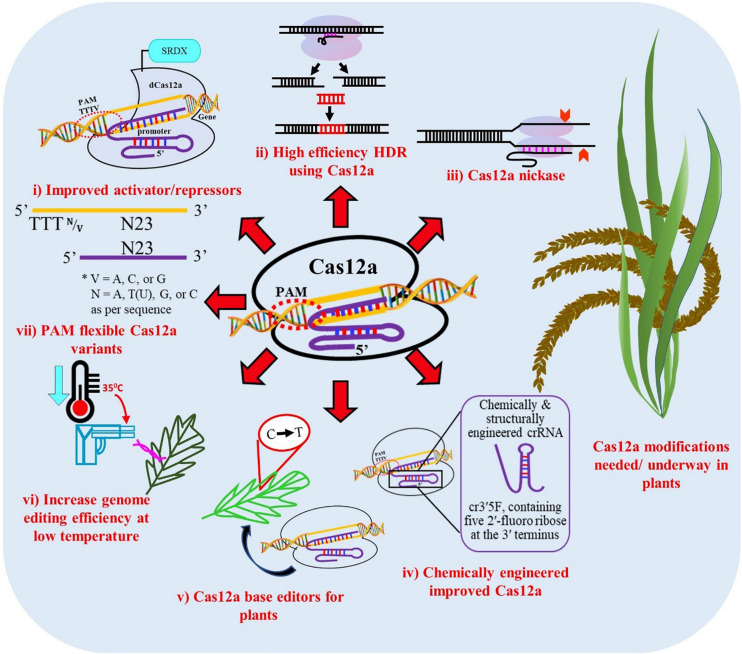
Proposed modifications of Cas12a for improved editing efficiency in plants. **(i)** Improved dCas12a activators and repressors for modifying gene expression, **(ii)** High efficiency gene targeting through homology repair mechanisms, **(iii)** Nickases, **(iv)** Chemically modified and structurally engineered crRNA, **(v)** Base editors for point mutations and indel insertions, **(vi)** Increase genome editing efficiency at low temperature, **(vii)** PAM-flexible Cas12a variants.

As mentioned above, there are numerous advantages to utilizing an HDR-dependent pathway to engineer alleles of interest. In particular, because a template is provided, this template can be synthesized to contain naturaly occurring or novel alleles of any given locus. However, as shown in Table 4, the efficiencies of HDR are quite variable relative to target loci and both Cas12a and Cas9 have been used successfully for HDR. Various approaches and vector construct designs have been used to direct homology-dependent repair pathway utilizing the CRISPR-Cas12a endonuclease system. In one of the first examples of Cas12a-mediated gene replacements in plants, 1 kb of homologous sequence flanking a target sequence was used to insert a selectable marker into the *Chlorophyllide-a oxygenase* (*OsCAO*) locus in rice. Reagents including LbCas12a and FnCas12a plasmids, donor template and the crRNA expression construct were introduced as DNA templates through particle bombardment and insertion events identified (Begemann et al., 2017). Similar frequencies of HDR (4.6–7%) were obtained in the zebrafish model system when reagents were delivered as ribonucleoproteins coupled with donor template DNA. In this example, LbCas12a mediated homologous gene replacement at target loci *slc45a2* (albino) and *tyr* (tyrosinase) at higher efficiency than SpCas9 (Moreno-Mateos et al., 2017). Li et al. (2018a) also utilized RNP delivery but used RNA templates to mediate the HDR of ALS. Further refinements to the Cas12a system including the utilization of ribozymes and silent PAMs for homologous gene replacement in rice and maize have increased the frequency of template mediated repair using LbCas12a (Wolter and Puchta, 2019; Li et al., 2019b). To increase the availability of donor template van Vu et al., 2020 utilized a geminivirus replicon system to introduce a salt tolerance allele of *ANT1* and achieved a higher HDR efficiency rate of 9.8% compared with SpCas9 in tomato. Application of various strategies to insert genes through a homologous repair pathway enables one to edit crops with desired traits at a high frequency which is otherwise not possible with standard transgenic approaches. Further improvement and novel strategies of improving homologous recombination is greatly needed to fulfill important need of allele replacement in higher crops. Multiple techniques need to be tested widely such as combining different Agrobacterium virulence proteins (*Vir* proteins) with Cas12a, recruiting HDR proteins such as RAD group proteins with Cas12a, increasing donor template concentration in the presence of DSB by viral vectors (Figure 5).

## Summary

The utility of genome editing in plants is clear. From deepening our understanding of fundamental biological processes to engineering synthetic circuits and potentially introducing entirely novel biosynthetic pathways into production hosts, the CRISPR-Cas toolbox truly is revolutionizing plant biology. Here, we have reviewed some of the fundamental differences between the two most widely utilized systems for plant genome engineering and suggest that both Cas9 and Cas12a have unique advantages and disadvantages for genome engineering. As off target mutations occur at low frequencies with both enzymes (Stupar et al., 2020), it is recommended that seed stocks should not be maintained with active Cas proteins in the genome. In the case of RNP delivery, this is not an issue, but when the reagents are delivered as DNA molecules, it is likely that the frequency of off targets will increase the longer the nuclease remains in the genome and primary targets are exhausted. Once the primary target is mutated and no longer serves as a target for the gRNA, then the probability of the gRNA identifying a new target even with some mismatches likely increases. In practice, and in particularly for plant breeding applications, these potential off-target events would be purged with successive backcrossing and in light of alternative approaches (e.g., chemical mutagenesis), the mutational load associated with CRISPR/Cas will be dramatically lower. Nevertheless, strategies to induce and characterize CRISPR-induced alleles, should incorporate the segregation of the CRISPR transgenes out of the plant genome and ensure that alleles generated are homozygous rather than chimeric/heterozygous.

Future strategies to develop synthetic circuits (Jusiak et al., 2016) or to engineer novel pathways will likely incorporate multiple Cas enzymes that can serve alternatively as repressors or activators of suites of genes (Lowder et al., 2018; Ming et al., 2020). These artificial transcriptional activators or repressors can be guided to specific loci to globally up or down regulate entire suites of genes. It is also easy to envision scenarios where entirely orthologous circuits are introduced and regulated by entirely novel promoter elements. In such a way an entirely new pathway may be introduced and expressed in a developmentally- or environmentally controlled manner.

Although the possibilities of engineering plant systems are exciting, these strategies must also be tempered by the regulatory environment that exists. As with any new technologies the potential benefits will be weighed against the potential risks of the technology. Many in the agricultural industry hope that the development of traits that will directly benefit the consumer will help drive public acceptance of the technology. However, diverse stakeholders and special interest groups who benefit from the fractionation of genome editing technologies into clearly defined buckets (e.g., GMO and non-GMO), will likely oppose the technology no matter how low the risk or big the benefit as we have witnessed with GMO technologies. Thus, it will be critical to establish sound and transparent regulatory frameworks for genome editing technologies and for scientists to not only be good stewards of the technologies but to actively participate in public forums to discuss the technology.

## Author Contributions

AB and SD conceptualized the idea. NK, TB, and AB wrote, reviewed, and edited the manuscript. VJ and NK drew the images. NK tabulated the tables. All authors contributed to the article and approved the submitted version.

## Conflict of Interest

TB is employed by Gateway Biotechnology, Inc. The remaining authors are employed by Reliance Industries Ltd. TB and all other authors ensure that the research was conducted in the absence of any commercial or financial relationships that could be construed as a potential conflict of interest.
